# Inhibition of Lipoprotein-Associated Phospholipase A2 Ameliorates Inflammation and Decreases Atherosclerotic Plaque Formation in ApoE-Deficient Mice

**DOI:** 10.1371/journal.pone.0023425

**Published:** 2011-08-31

**Authors:** Wen-yi Wang, Jie Zhang, Wen-yu Wu, Jie Li, Yan-ling Ma, Wei-hai Chen, Hong Yan, Kai Wang, Wen-wei Xu, Jian-hua Shen, Yi-ping Wang

**Affiliations:** 1 Department of Pharmacology I, State Key Laboratory of Drug Research, Shanghai Institute of Materia Medica, Chinese Academy of Sciences, Shanghai, China; 2 Key Laboratory of Cognition and Personality (SWU), Ministry of Education, Southwest University, Chongqing, China; 3 School of Psychology, Southwest University, Chongqing, China; 4 Drug Discovery and Design Center, State Key Laboratory of Drug Research, Shanghai Institute of Materia Medica, Chinese Academy of Sciences, Shanghai, China; Istituto Dermopatico dell'Immacolata, Italy

## Abstract

**Background:**

Lipoprotein-associated phospholipase A2 (Lp-PLA2) is thought to play modulatory roles in the development of atherosclerosis. Here we evaluated the effects of a specific lp-PLA2 inhibitor on atherosclerosis in ApoE-deficient mice and its associated mechanisms.

**Methodology/Principal Findings:**

ApoE-deficient mice fed an atherogenic high-fat diet for 17 weeks were divided into two groups. One group was administered the specific lp-PLA2 inhibitor, darapladib (50 mg/kg/day; p.o.) daily for 6 weeks, while the control group was administered saline. We observed no differences in body weight and serum lipids levels between the two groups at the end of the dietary period. Notably, serum lp-PLA2 activity as well as hs-CRP (C-reactive protein) and IL-6 (Interleukin-6) levels were significantly reduced in the darapladib group, compared with the vehicle group, while the serum PAF (platelet-activating factor) levels were similar between the two groups. Furthermore, the plaque area through the arch to the abdominal aorta was reduced in the darapladib group. Another finding of interest was that the macrophage content was decreased while collagen content was increased in atherosclerotic lesions at the aortic sinus in the darapladib group, compared with the vehicle group. Finally, quantitative RT-PCR performed to determine the expression patterns of specific inflammatory genes at atherosclerotic aortas revealed lower expression of MCP-1, VCAM-1 and TNF-α in the darapladib group.

**Conclusions/Significance:**

Inhibition of lp-PLA2 by darapladib leads to attenuation of *in vivo* inflammation and decreased plaque formation in ApoE-deficient mice, supporting an anti-atherogenic role during the progression of atherosclerosis.

## Introduction

Atherosclerosis is the most common cause of cardiovascular diseases, such as myocardial infarction and stroke. The development of atherosclerosis is associated with both lipids metabolism and inflammation [1].

Lp-PLA2, also designated platelet-activating factor acetylhydrolase (PAF-AH; E.C. 3.1.1.47), is a special Ca^2+^-independent phospholipase associated mainly with apoB-containing lipoproteins and primarily with LDL in humans [2]. Epidemiological studies have suggested that elevated circulating lp-PLA2 is predictive of increased cardiovascular risk [3]. Lp-PLA2 is up-regulated by oxidized phospholipids in oxLDL, and in turn, acts on these oxidized phospholipids (oxPCs) to produce two pro-inflammatory mediators, lysophosphatidylcholines (lysoPCs) and oxidized non-esterified fatty acids (oxNEFAs) [4]. There is considerable evidence to support regulatory roles of these two products, particularly lysoPCs, in promoting atherosclerotic plaque development. For instance, lysoPCs can recruit leukocytes to lesions, activate leukocytes to initiate immune responses, and promote foam cell formation [5], [6].

The *in vitro* and *ex vivo* findings collectively suggest a causative role of lp-PLA2 in the development of atherosclerosis, and inhibition of its activity may thus induce beneficial effects. An earlier study reported that the lp-PLA2 inhibitor, darapladib, reduces complex coronary atherosclerotic plaque development in pigs with induced diabetes and hypercholesterolemia [7]. Darapladib has additionally shown beneficial effects in clinical studies. The compound attenuates the inflammatory burden in patients with stable coronary heart disease and prevents necrotic core expansion, a key determinant of plaque vulnerability [8].

However, no *in vivo* data are available on the effects of the lp-PLA2 inhibitor on atherosclerosis development in mouse models. This may be due to two reasons. Firstly, the lipid profile and distribution of lp-PLA2 in plasma of mice are distinct from those in humans [9]. Lp-PLA2 is mainly associated with HDL in mice and LDL in humans, and its role in atherosclerosis may thus differ, depending on the lipoprotein carrier in plasma [10]. Secondly, other *in vivo* experiments in mice have suggested an anti-atherogenic role of lp-PLA2. Specifically, adenovirus-mediated gene transfer of human lp-PLA2 prevented injury-induced neointima formation and reduced spontaneous atherosclerosis in ApoE-deficient mice as well as accumulation of oxidized lipoproteins, and inhibited inflammation and thrombosis in non-hyperlipidemic rabbits [11], [12].

In this study, we examined the effects of the lp-PLA2 inhibitor, darapladib, in ApoE-deficient mice to further establish its role in development of atherosclerosis.

## Materials and Methods

### Animals and experimental protocol

Male homozygous ApoE-deficient mice (C57/Bl6 genetic background) were obtained from the Jackson Laboratory. The following protocols were approved by the Animal Care and Use committee of the Shanghai Institute of Materia Medica, Chinese Academy of Sciences (Approval ID: SIMM-AE-WYP-2010-03).

The cages (length is 280 mm, width is 165 mm, high is 135 mm) with toys inside were used to contained less than 5 mice, all animals were housed in a temperature (21–26°C), humidity (40–70%), and light-cycle controlled( Light was on between 7:00 am and 7:00 pm) room. Mice were fed a high-fat diet (containing 18% hydrogenated cocoa butter, 0.15% cholesterol, 7% casein, 7% sucrose, and 3% maltodextrin) for 17 weeks, starting at 6 weeks of age. Fifty mice were divided into two groups randomly (N = 25 per group). One group received darapladib (50 mg/kg/. p.o.) once daily, while the other group received vehicle. During the 6 weeks of treatment, all mice allowed free access to a high-fat diet and water.

### Serum lipid analysis

Mice were anesthetized slightlyby diethyl ether when blood samples were obtained from the retroorbital plexus before drug administration and 24 hours after the last drug administration. Serums were acquired through centrifugation of the blood samples at 4°C at 1000 g, and stored at −80°C until analysis. Total cholesterol (TC), high-density lipoprotein cholesterol (HDL-C), low-density lipoprotein cholesterol (LDL-C) and triglyceride (TG) levels were measured enzymatically with commercial kits from Wako Inc. using an auto-analyzer (Hitachi 7100, Japan).

Serum lp-PLA2 activity was measured using 2-thio-PAF as the substrate. Ten µL of serum was added to 0.1 M Tris-HCl (PH 7.2) containing 1 mM EGTA, 50 µM 2-thio-PAF and 10 µL of 2 mM 5,5′-dithio-bis-(2-nitrobenzoic acid) in a total volume of 200 µL. The assay was performed using a plate reader to obtain absorbance values at 414 nm every minute. The lp-PLA2 activity was calculated from the change in absorbance per minute (Cayman Chem. Lot 76091).

Serum IL-6, hs-CRP, and PAF levels were determined by ELISA according to manufacturers' instructions (Mingrui Biotech. Inc.).

### Morphology of atherosclerotic plaques

After 6 weeks of treatment, mice were anesthetized by diethyl ether and sacrificed. The mice were dissected and aortas were perfusion-fixed with 4.5% formaldehyde. Then the aortas were dissected, from the heart to approximately 3 mm distal to the iliac bifurcation. The aortas were preserved in fresh paraformaldehyde solution for 2 weeks and Sudan IV staining was employed to determine the plaques on entire aortas. Briefly, after removing surrounding adventitial fatty tissue, the aortas were opened longitudinally and pinned out on a black silica gel plate. The aorta was rinsed in 70% ethanol after 12 hours of fixation in the paraformaldehyde solution, stained with 1% Sudan IV in 50% acetone/35% ethanol for about 10 minutes, and washed in 80% ethanol for 5 minutes. Finally, the stained aortas were photographed and analyzed using the Image Pro-Plus 6.0 software.

### Immunohistochemistry

Paraffin sections through the aortic sinus were obtained and used to quantify the lesion composition. Briefly, paraffin sections were deparaffinized, rehydrated, and subsequently treated with 0.3% H_2_O_2_ for 10 minutes to abolish endogenous peroxidase activity, and 5% BSA for 1 hour to block non-specific antibody binding. Subsequently, sections were incubated overnight at 4°C with murine smooth muscle actin antibody (1∶50 dilution, Beyotime Biotech Inc.) or Mac-2 antibody (1∶50 dilution, Santa Cruz Inc.), respectively. After washing, sections were incubated with secondary antibodies for 1 hour at room temperature and finally visualized with a DAB kit (Beyotime Biotech Inc.). For collagen determination, Masson trichrome staining was performed, according to the manufacturer's instructions (Genmed Inc.).

### Quantitative RT-PCR

After 6 weeks of treatment, total RNA was extracted from the aortic arch and thoracic-abdominal aortas of the two groups of mice using TRIZOL reagent (Invitrogen Inc.). First-strand cDNAs were synthesized from 4 µg of total RNA using M-MLV reverse transcriptase (Promega Inc.). Quantitative real-time PCR was performed using SYBR Green I as the detector dye. Relative gene expression was calculated by normalizing to the quantity of mouse actin gene. Primers' sequences are shown in Table S1.

### Statistical analysis

Data are presented as mean values ± SEM. For serum lipids and lp-PLA2 activities, comparisons were made using one-way analysis of variance (ANOVA), followed by the post-hoc Dunnett test for significance. Comparisons of body weight, plaque area and gene expression were made using the two-tailed Student's t-test. For all tests, P<0.05 was considered statistically significant.

## Results

### Darapladib inhibits mouse serum lp-PLA2 activity *in vivo*

**Figure 1:**
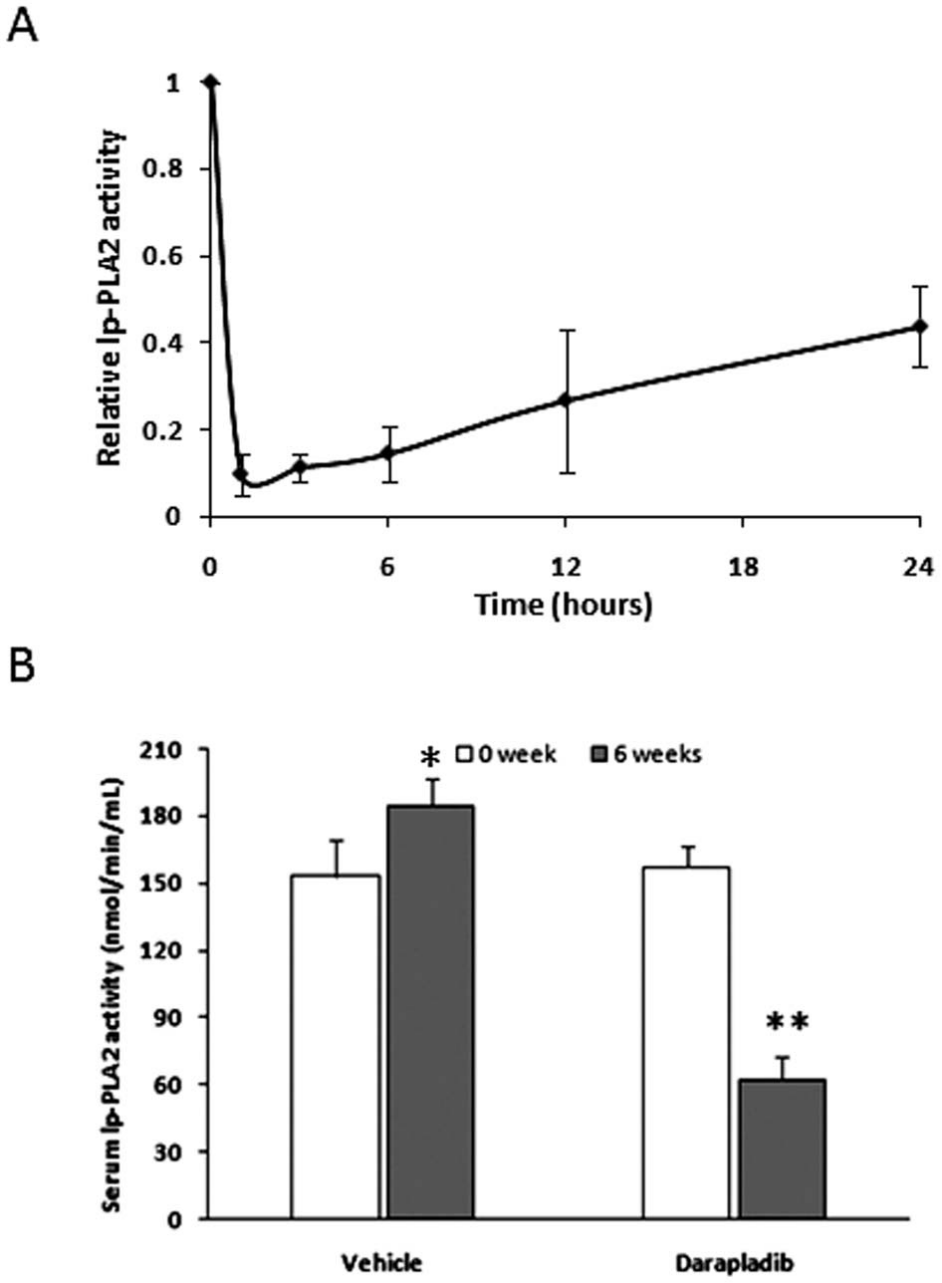
Darapladib significantly inhibits serum lp-PLA2 activity in ApoE-deficient mice. (A). Five apoE-deficient mice were administrated with darapladib (50 mg/kg p.o.) and serum lp-PLA2 activity before or 1, 3, 6, 12, 24 hours after administration was measured. (B). Serum lp-PLA2 activity was measured using spectrometry before and at the end of drug administration. **p<0.01 vs. vehicle at 6 weeks. and *p<0.05 vs. vehicle at 0 week.

We examined a range of darapladib concentrations to determine the dose inducing inhibition of mouse plasma lp-PLA2 before continuous administration, and found the dosage of 50 mg/kg.p.o inhibits the plasma lp-PLA2 activity remarkably (Figure 1A). Thus, the dose was chose to administrate the ApoE-deficient mice during the whole periods. As shown in Figure 1B, plasma lp-PLA2 activity was inhibited by more than 60% after oral administration of 50 mg/kg of darapladib once daily for 6 weeks.

### Inhibition of lp-PLA2 has no significant effects on the serum lipid profile

The serum lipid level and body weight were evaluated in both groups. As expected, we observed no significant differences in the TC, TG, LDL-C and HDL-C levels between vehicle and darapladib-treated ApoE-deficient mice. Additionally, the body weights of ApoE-deficient mice in both groups were similar (Table 1).

**Table 1:** Effects of inhibition of lp-PLA2 by darapladib on body weight (g), serum total cholesterol, triglyceride, HDL-C and LDL-C levels (mM) in ApoE-deficient mice.

	Body weight	Total cholesterol	Triglyceride	HDL cholesterol	LDL cholesterol
Vehicle	31.6±0.7	45.44±1.49	1.70±0.06	2.30±0.15	36.67±1.53
Darapladib	30.1±0.9	48.19±2.01	1.74±0.12	2.48±0.15	37.40±2.00

N = 25 per group.

### Inhibition of lp-PLA2 triggers decreases in serum inflammatory markers

**Figure 2:**
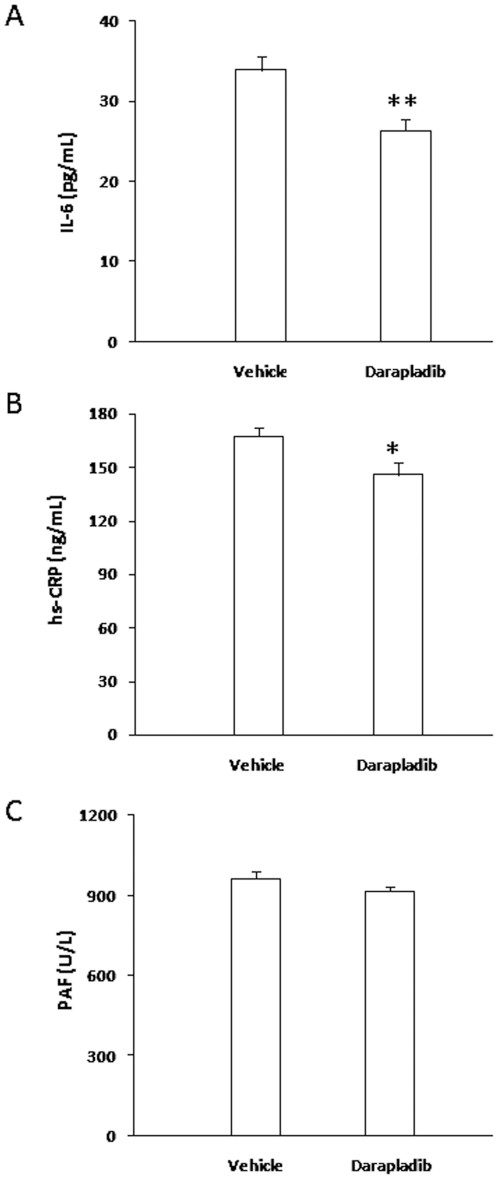
Inhibition of lp-PLA2 by darapladib decreases serum hs-CRP and IL-6 levels, but has no significant effects on the PAF level. After 6 weeks of feeding, serum hs-CRP, IL-6 and PAF levels were determined using ELISA. *p<0.05 and **p<0.01 vs. vehicle.

Lp-PLA2 is considered a novel anti-atherosclerosis target from the inflammatory viewpoint. Accordingly, we evaluated the serum levels of two typical inflammatory markers, hs-CRP and IL-6, using ELISA. Levels of both hs-CRP and IL-6 were significantly reduced in the darapladib group, compared with those in the vehicle group of ApoE-deficient mice (Figures 2A and 2B). As mentioned above, lp-PLA2, also named PAF-AH, hydrolyzes PAF, a strong inflammatory mediator, and may thus exert an anti-inflammatory effect. To examine this theory, we determined the effects of darapladib on the serum PAF level. As shown in Figure 2C, no significant differences were evident in the serum PAF levels between the two groups.

### Inhibition of lp-PLA2 significantly reduces the formation of atherosclerotic lesions

**Figure 3:**
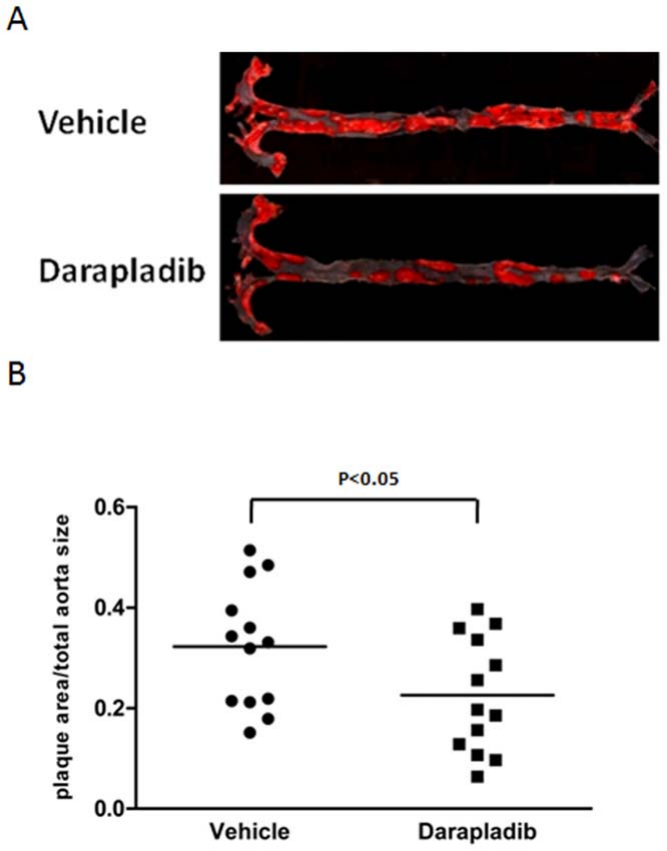
Inhibition of lp-PLA2 decreases the atherosclerotic area. (A). Representative en face atherosclerotic aorta preparations stained with Sudan IV. (B). Comparison of plaque sizes between the vehicle and darapladib groups (N = 13 per group). The area stained with dye is expressed as a percentage of the total surface area. The mean is depicted as a single horizontal line.

To ascertain the effects of the lp-PLA2 inhibitor on atherosclerotic lesion formation, we detected plaque sizes at the proximal aorta via Sudan IV staining. As shown in Figure 3, inhibition of lp-PLA2 induced a significant decrease in the plaque area. Vehicle-treated mice displayed approximately 32±3% plaque coverage, whereas mice treated with darapladib had 22±3% plaque coverage (Figure 3B).

**Figure 4:**
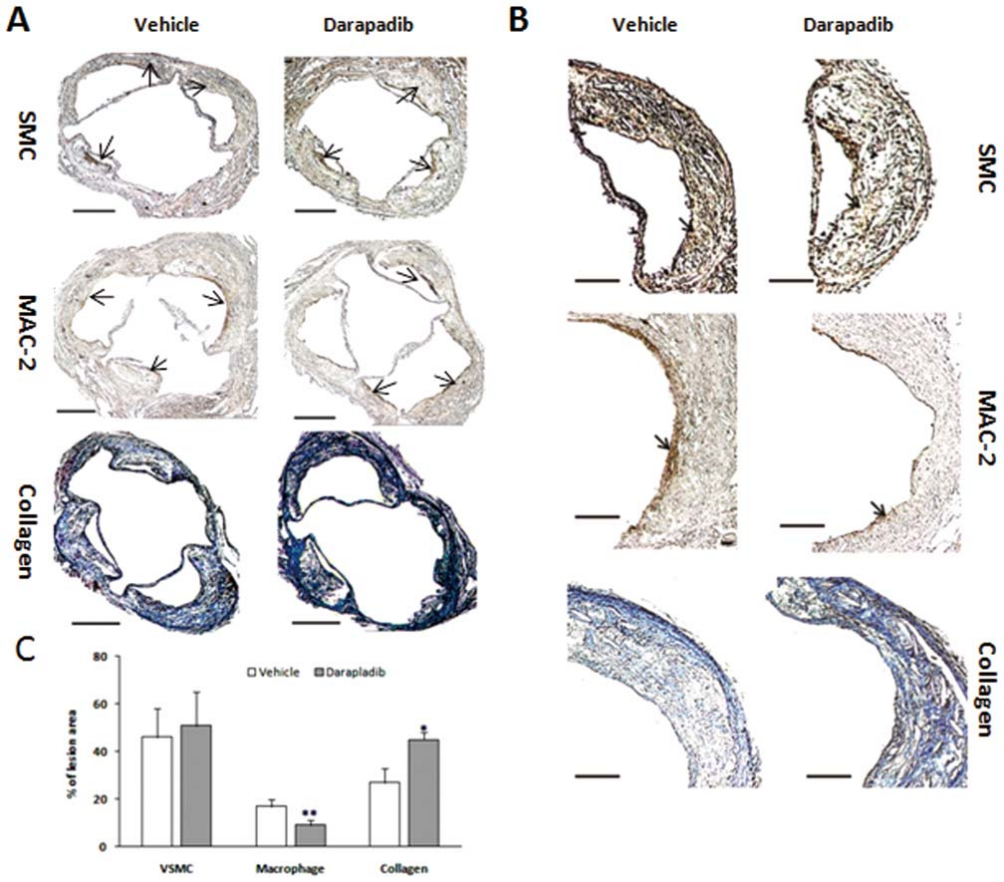
Composition of atherosclerotic plaques in the aortic sinus. (A) and (B). Representative examples are provided for VSMC-Actin, macrophage antibody (Mac-2) and trichrome-stained aortic sinus sections. Arrows are indicative of representative regions staining positively for VSMCs and macrophages. The collagen regions are indicated in blue. The bar indicates 400 µm (A) and 200 µm (B), respectively. (C). Comparison of VSMC, macrophage and collagen content between the two groups (N = 7 per group), *p<0.05 and **p<0.01 vs. vehicle.

To evaluate lesion composition, we immunostained the lesions for macrophages, VSMCs, as well as collagen content. The macrophage content in lesions of the darapladib group was lower and the collagen content higher, compared with the vehicle group. However, the VSMC content was shown no significant difference between the two groups (Figure 4).

### Inhibition of lp-PLA2 attenuates inflammatory gene expression at the lesion areas

**Figure 5:**
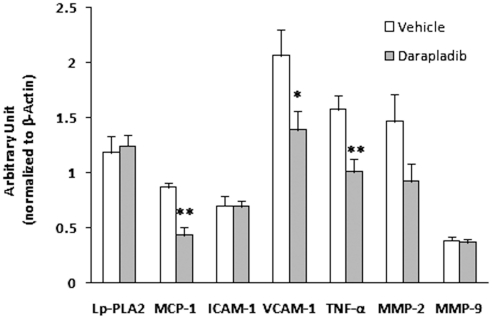
Inhibition of lp-PLA2 attenuates inflammatory gene expression at the aortic arch and thoracic-abdominal aortas. Lp-PLA2, MCP-1, ICAM-1, VCAM-1, TNF-α, MMP-2 and MMP-9 gene expression was determined using quantitative RT-PCR. N = 5 per group, *p<0.05, and **p<0.01 vs. vehicle.

We additionally evaluated the expression of lp-PLA2 and several inflammatory genes in the arteries using quantitative realtime PCR. As shown in Figure 5, lp-PLA2 gene expression was not significantly different between the darapladib and vehicle groups. However, several inflammatory genes, MCP-1, VCAM-1 and TNF-α, were remarkably reduced in the darapladib group, compared with the vehicle group. Unexpectedly, ICAM-1, MMP-2 and MMP-9 expression patterns were comparable between the two groups (Figure 5).

## Discussion

Atherosclerosis contributes to myocardial infarction and stroke, the principal causes of death in developed societies. Dyslipidemia and inflammation cooperate to promote atherosclerosis progression. Statins, widely used in the treatment of atherogenic dyslipidemia, exert significant beneficial effects on coronary heart disease, but do not completely diminish cardiovascular risks [13]. Thus, other targets independent of dyslipidemia are urgently required to decrease cardiovascular risk. The aim of this study was to evaluate the effects of a specific inhibitor of the new therapeutic target, lp-PLA2, on atherosclerosis development in high-fat diet fed mice.

The lp-PLA2 inhibitor has been shown to reduce atherosclerostic plaque formation in diabetes mellitus and hypercholesterolemia (DM-HC) pigs [7]. Darapladib treatment resulted in a considerable decrease in the plaque and necrotic core areas. Inhibition of lp-PLA2 also induced beneficial effects in clinical research, as darapladib terminated the increase in necrotic core, a key determinant of plaque vulnerability, despite having no effect on plaque formation [8].

ApoE-deficient mice have been extensively used to evaluate the efficacy of anti-atherogenic drugs and the mechanisms of atherosclerosis progression. As the uptake of lipoprotein particles is impaired, these mice spontaneously display elevated blood lipid levels. The high-fat diet leads to increased cholesterol and TG levels in both VLDL and LDL fractions, as well as lesion formation. High VLDL and LDL or low HDL levels are known risk factors of cardiovascular disease. However, treatment with the lp-PLA2 inhibitor did not alter the plasma lipoprotein levels or profile. In another study, adenovirus-mediated gene transfer of lp-PLA2 significantly enhanced the lp-PLA2 level without changing the lipid profile in mice [11]. Thus, lp-PLA2 exerts a modulatory role in the progression of atherosclerosis, independent of the lipoprotein profile.

Several epidemiological studies have shown that the plasma level and activity of lp-PLA2 increase during the development of atherosclerosis, supporting its utility as a marker for cardiovascular risk [14], [15], [16], [17], [18]. In the current study, we additionally demonstrated that the serum lp-PLA2 activity increased in the vehicle group after high-fat feeding for 6 weeks, but was significantly inhibited in the darapladib group.

Lp-PLA2 is thought to play an important modulatory role in atherogenesis due to its catalytic activity in hydrolysis of bioactive lipids, such as PAF and oxPCs. However, the precise role of lp-PLA2 is controversial. PAF is a typical pro-inflammatory factor that contributes to tissue damage and thrombosis formation, among other effects [19]. From this viewpoint, lp-PLA2 would exert an anti-atherogenic role by inactivating PAF. However, there is no evidence that lp-PLA2 hydrolyzes PAF *in vivo*. In the present study, inhibition of lp-PLA2 by darapladib did not affect the serum PAF level. In addition, intravenous administration of recombinant lp-PLA2 failed to alter PAF-mediated responses in patients with asthma or septic shock [20], [21].

Conversely, considerable evidence has been obtained for pro-atherogenic roles of lp-PLA2 *in vitro* and *in vivo* [2], [7], [22], [23]. Lp-PLA2 produces two types of inflammatory mediators, lysoPCs and oxNEFA, via hydrolyzing oxidized phospholipids in oxLDL, which trigger significant inflammatory responses, such as cell adhesion, inflammatory gene expression, and cell death [5], [24]. Furthermore, *in vivo* studies have suggested that inhibition of lp-PLA2 by darapladib decreases the inflammatory burden in humans and pigs [7], [8], [23], [25]. Here, we examined the inflammatory factors in serum and gene expression of inflammatory cytokines in lesions. Consistent with earlier *in vivo* findings, the inflammatory burden decreased in the darapladib group, compared with that in the vehicle group. Along with previous reports, our present findings support a pro-inflammatory role of lp-PLA2 *in vivo*.

The present study has some limitations, as the detailed mechanisms underlying the anti-atherogenic effects of the lp-PLA2 inhibitor remain unclear. Based on the previous and present data, we suggest that the beneficial effects of darapladib against the formation of atherosclerosis are possibly attributed to its anti-inflammation properties. In clinical research, oral darapladib (160 mg once daily) induced a significant decrease in plasma IL-6 and hs-CRP levels [25]. Similarly, in another clinical report, inhibition of lp-PLA2 suppressed the hs-CRP level [8]. Furthermore, analysis of coronary gene expression revealed that inhibition of lp-PLA2 led to a substantial reduction in the expression of many genes associated with macrophage and T lymphocyte functioning in pigs [7]. In the present investigation, inhibition of lp-PLA2 attenuated the inflammatory burden in ApoE-deficient mice, as evident from the decreased serum levels of hs-CRP and IL-6 and inflammatory gene expression in the darapladib-treated group. LysoPCs, the products of lp-PLA2 inducing many inflammatory genes expression has been well established. For instance, lysoPCs can up-regulate MCP-1 and ICAM-1/VCAM-1 expression in endothelial cells or VSMCs [6], [26], [27], [28]. It is apparent that inhibition of lp-PLA2 may decrease arterial lysoPCs abundance and consequent reduction of inflammatory burden. We did not measure the lysoPCs in mice aortas in present study; nevertheless, darapladib treatment was associated with a decrease in elevated arterial lysoPCs abundance in DM-HC pigs [7]. However, the possibility that darapladib exerts its anti-atherosclerosis effects through other mechanisms cannot be excluded. For instance, the compound may directly reduce the uptake of lipids by macrophages, as demonstrated in a previous study by our group [4]. The beneficial effects may also be induced by the compound itself, independent of lp-PLA2. Further studies are essential to investigate the precise mechanisms by which lp-PA2 inhibition triggers anti-atherosclerosis effects.

Another noteworthy finding is that the collagen content at the lesion area in the darapladib-treated group was higher than that of the vehicle group. Vascular remodeling, especially extracellular matrix (ECM) increase, is thought to stabilize plaques, which may prevent disruption of lesions [29], [30]. Based on the present data, we propose that darapladib increases the collagen content or decreases degradation of collagen in the lesion areas, indicating that inhibition of lp-PLA2 stabilizes plaques, consistent with previous results [31]. As MMPs play pivotal roles in ECM degradation, we determined MMP-2 and MMP-9 expression within the lesion areas. Interestingly, no differences were observed in the MMP expression patterns between the vehicle and darapladib groups. However, MMP-2 activity was lower at the lesion area in the darapladib group (unpublished data). Further studies are needed to explore the interplay between lp-PLA2 and MMPs.

In summary, our *in vivo* studies demonstrate that inhibition of lp-PLA2 by darapladib does not decrease dyslipidemia but ameliorates the inflammatory burden, resulting in decrease of atherosclerosis in high-fat diet-fed ApoE-deficient mice. Our present study validates the feasibility of anti-inflammation therapeutic strategies for the effective treatment of cardiovascular disease and the utility of lp-PLA2 as a promising target against atherosclerosis.

## Supporting Information

Table S1 Gene primer sequences for quantitative real-time PCR.
(DOC)
