# Supplementary material for: Inhibition of Lipoprotein-Associated Phospholipase A2 Ameliorates Inflammation and Decreases Atherosclerotic Plaque Formation in ApoE-Deficient Mice
Source: PLoS One. 2011 Aug 31;6(8):e23425. doi: 10.1371/journal.pone.0023425 (PMC3166130; doi:10.1371/journal.pone.0023425)
Supplement: Table S1 — Gene primer sequences for quantitative real-time PCR. (DOC) [file pone.0023425.s001.doc]

| **Gene** | **Forward** | **Reverse** | **Genebank ID** |
| --- | --- | --- | --- |
| Lp-PLA2 | GAGCGTCTTCGTGCGTTTG | GCGGGTATTTTTCTCCAGTC | NM_013737 |
| MCP-1 | CCTGCTGTTCACAGTTGCC | TGTCTGGACCCATTCCTTCT | NM_011333 |
| ICAM-1 | GCTGTATGGTCCTCGGCTG | GCCCACAATGACCAGCAGTA | NM_010494 |
| VCAM-1 | TGAACCCAAACAGAGGCAGA | CGGAATCGTCCCTTTTTGTAG | NM_011693 |
| MMP-2 | CAAAGAAAGGTGCTGACTGT | GAAGGAAACGAGCGAAGG | NM_008610 |
| MMP-9 | CAGCCAACTATGACCAGGAT | TGCCGTCTATGTCGTCTTTA | NM_013599 |
| TNF-α | GGGCAGGTCTACTTTGGAG | AGCCCATTTGAGTCCTTGAT | NM_013693 |
| β-Actin | GGGAAATCGTGCGTGACA | CAAGAAGGAAGGCTGGAAAA | NM_007393 |
